# Complete genome sequence of the bacteriochlorophyll *a*-containing *Roseibacterium elongatum* type strain (DSM 19469^T^), a representative of the *Roseobacter* group isolated from Australian coast sand

**DOI:** 10.4056/sigs.5541028

**Published:** 2014-03-28

**Authors:** Thomas Riedel, Anne Fiebig, Markus Göker, Hans-Peter Klenk

**Affiliations:** 1 Sorbonne Universités, UPMC Univ Paris 06, USR3579, LBBM, Observatoire Océanologique, Banyuls/Mer, France; 2 CNRS, USR3579, LBBM, Observatoire Océanologique, Banyuls/Mer, France; 3 Helmholtz Centre for Infection Research, Braunschweig, Germany; 4 Leibniz Institute DSMZ – German Collection of Microorganisms and Cell Cultures, Braunschweig, Germany

**Keywords:** bacteriochlorophyll *a*, aerobic anoxygenic photosynthesis, quorum sensing, *Roseobacter* group, *Roseibacterium*, *Rhodobacteraceae*, *Alphaproteobacteria*

## Abstract

*Roseibacterium elongatum* Suzuki *et al*. 2006 is a pink-pigmented and bacteriochlorophyll *a*-producing representative of the *Roseobacter* group within the alphaproteobacterial family *Rhodobacteraceae*. Representatives of the marine ‘*Roseobacter* group’ were found to be abundant in the ocean and play an important role in global and biogeochemical processes. In the present study we describe the features of *R. elongatum* strain OCh 323^T^ together with its genome sequence and annotation. The 3,555,102 bp long genome consists of one circular chromosome with no extrachromosomal elements and is one of the smallest known *Roseobacter* genomes. It contains 3,540 protein-coding genes and 59 RNA genes. Genome analysis revealed the presence of a photosynthetic gene cluster, which putatively enables a photoheterotrophic lifestyle. Gene sequences associated with quorum sensing, motility, surface attachment, and thiosulfate and carbon monoxide oxidation could be detected. The genome was sequenced as part of the activities of the Transregional Collaborative Research Centre 51 (TRR51) funded by the German Research Foundation (DFG).

## Introduction

Strain OCh 323^T^ (= DSM 19469^T^ = CIP 107377^T^ = JCM 11220^T^) is the type strain of *Roseibacterium elongatum* in the bispecific genus *Roseibacterium* [1] with *R. beibuensis* [2] being the second species in the genus. The genus *Roseibacterium* belongs to the marine *Roseobacter* group, which was shown to be ubiquitious in the oceans of the world, especially in coastal and polar oceans [3,4]. The strain was isolated from sand located at Monkey Mia, Shark Bay, at the west coast of Australia [1]. The genus *Roseibacterium* was named after the Latin adjective *roseus* (‘rose, pink’) and the Greek adjective bakterion (‘rod’); *Roseibacterium* (‘pink, rod-shaped bacterium’). The species epithet *elongatum* refers to the Latin adjective *elongatum* (‘elongated, stretched out’) [1]. Current PubMed records do not indicate any follow-up research with strain OCh 323^T^ after the initial description of *R. elongatum* [1].

In this study we analyzed the genome sequence of *R. elongatum* DSM 19469^T^. We present a description of the genome sequencing and annotation and a summary classification together with a set of features for strain DSM 19469^T^, including novel aspects of its phenotype.

## Features of the organism

### 16S rRNA gene analysis

**Figure 1:**
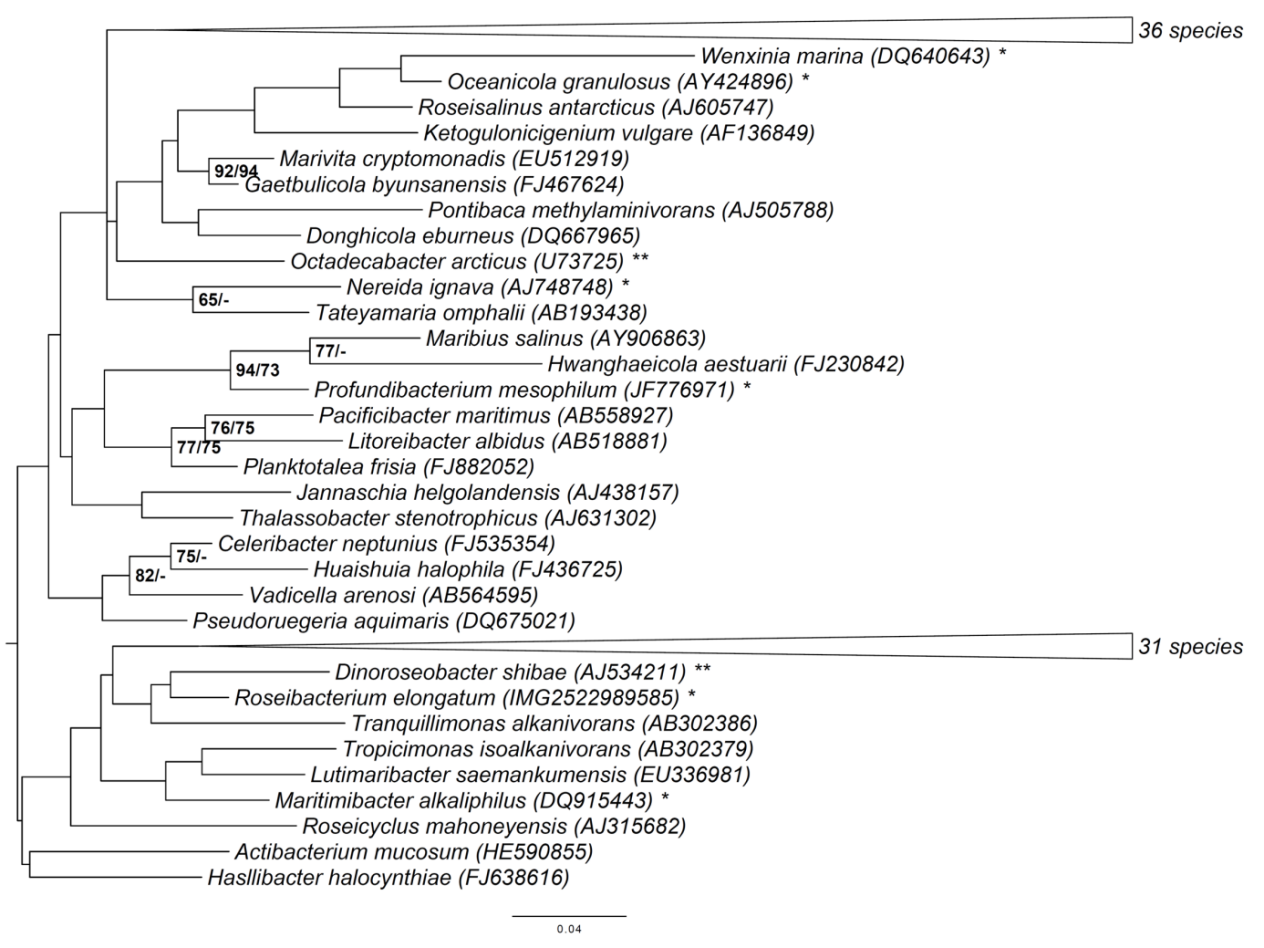
Phylogenetic tree highlighting the position of *R. elongatum* relative to the type strains of the type species of the other genera within the family *Rhodobacteraceae*. The tree was inferred from 1,331 aligned characters of the 16S rRNA gene sequence under the maximum likelihood (ML) criterion as previously described [5]. Rooting was done initially using the midpoint method [6] and then checked for its agreement with the current classification (Table 1). The branches are scaled in terms of the expected number of substitutions per site. Numbers adjacent to the branches are support values from 600 ML bootstrap replicates (left) and from 1,000 maximum-parsimony bootstrap replicates (right) if larger than 60% [5]. Lineages with type strain genome sequencing projects registered in GOLD [7] are labeled with one asterisk, those also listed as 'Complete and Published' with two asterisks [8-11].

Figure 1 shows the phylogenetic neighborhood of *R. elongatum* DSM 19469^T^ in a 16S rRNA gene based tree. The sequences of the two identical 16S rRNA gene copies in the genome do not differ from the previously published 16S rRNA gene sequence (AB601471).

**Table 1:** Classification and general features of *R. elongatum* OCh 323^T^ according to the MIGS recommendations [12] published by the Genome Standards Consortium [13].

**MIGS ID**	**Property**	**Term**	**Evidence code**
	Current classification	Domain *Bacteria*	TAS [14]
		Phylum *Proteobacteria*	TAS [15]
		Class *Alphaproteobacteria*	TAS [16,17]
		Order *Rhodobacterales*	TAS [17,18]
		Family *Rhodobacteraceae*	TAS [17,19]
		Genus *Roseibacterium*	TAS [1]
		Species *Roseibacterium elongatum*	
TAS [1]		Strain OCh 323^T^	TAS [1]
	Gram stain	negative	TAS [1]
	Cell shape	rod-shaped	TAS [1]
	Motility	non-motile	TAS [1]
	Sporulation	non-sporulating	NAS
	Temperature range	mesophile	NAS
	Optimum temperature	27-30°C	TAS [1]
	Salinity	0.5-7.5% NaCl	TAS [1]
MIGS-22	Oxygen requirement	aerobic	TAS [1]
	Carbon source	complex	NAS
	Energy metabolism	chemoheterotroph/photoheterotroph	TAS [1]
MIGS-6	Habitat	sand, seawater	TAS [1]
MIGS-15	Biotic relationship	free-living	TAS [1]
MIGS-14	Pathogenicity	none	NAS
	Biosafety level	1	TAS [20]
MIGS-23.1	Isolation	sand	TAS [1]
MIGS-4	Geographic location	Monkey Mia, Shark Bay, Australian west coast	TAS [1]
MIGS-5	Sample collection time	not reported	
MIGS-4.1	Latitude	-25.789	NAS
MIGS-4.2	Longitude	113.721	NAS
MIGS-4.3	Depth	not reported	
MIGS-4.4	Altitude	not reported	

Evidence codes - TAS: Traceable Author Statement (i.e., a direct report exists in the literature); NAS: Non-traceable Author Statement (i.e., not directly observed for the living, isolated sample, but based on a generally accepted property for the species, or anecdotal evidence). Evidence codes are from of the Gene Ontology project [21].

A representative genomic 16S rRNA gene sequence of *R. elongatum* DSM 19469^T^ was compared with the Greengenes database [22] for determining the weighted relative frequencies of taxa and (truncated [23]) keywords as previously described [5]. The most frequently occurring genera were *Rhodovulum* (35.1%), *Jannaschia* (13.5%), *Dinoroseobacter* (10.6%), *Rhodobacter* (9.6%) and *Roseobacter* (8.5%) (89 hits in total). Regarding the two hits to sequences from members of the species, the average identity within HSPs was 100.0%, whereas the average coverage by HSPs was 99.7%. Among all other species, the one yielding the highest score was *Dinoroseobacter shibae* (NC_009952), which corresponded to an identity of 95.7% and a HSP coverage of 100.1%. (Note that the Greengenes database uses the INSDC (= EMBL/NCBI/DDBJ) annotation, which is not an authoritative source for nomenclature or classification). The highest-scoring environmental sequence was AF513932 (Greengenes short name '*Rhodobacter* group clone LA4-B3'), which showed an identity of 99.4% and a HSP coverage of 99.9%. The most frequently occurring keywords within the labels of all environmental samples that yielded hits were 'microbi' (4.3%), 'mat' (2.3%), 'sea' (2.0%), 'marin' (2.0%) and 'coral' (1.9%) (157 hits in total). The most frequently occurring keywords within the labels of those environmental samples that yielded hits of a higher score than the highest scoring species were 'group, rhodobact' (33.8%) and 'rhodobacteracea' (32.4%) (2 hits in total). These keywords fit well to the known ecology (and phylogenetic relationships) of *R. elongatum* DSM 19469^T^.

### Morphology and physiology

**Figure 2:**
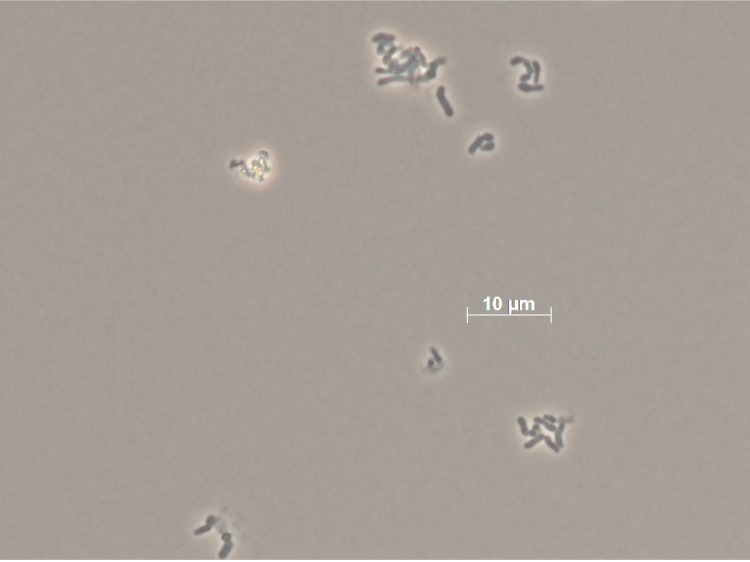
Micrograph of *R. elongatum* DSM 19469^T^.

Cells of strain OCh 323^T^ are Gram-negative, non-motile and rod-shaped, 1.6-10.0 µm in length and 0.5-0.8 µm in width (Figure 2). Colonies are circular, smooth, convex and glistening, opaque and pink-pigmented. Optimum growth occurs at a temperature of 27-30°C and a pH of 7.5-8.0. Cells can grow in the presence of 0.5-7.5% NaCl but do not grow in the absence of NaCl. Cells are positive for urease activity but do not show nitrate reductase or phosphate activities. They are negative in the Voges-Prosgauer test but the ONPG reaction is positive. Cells do not produce indole or H_2_S. Gelatin is hydrolyzed, but alginate, starch and Tween80 are not. Cells do not utilize acetate, citrate, D-glucose, DL-malate, ethanol, pyruvate, succinate. Acid is not produced from D-fructose, D-glucose or lactose (all data from [1]).

In this study the utilization of carbon compounds by *R. elongatum* DSM 19469^T^ grown at 28°C was also determined using Generation-III microplates in an OmniLog phenotyping device (BIOLOG Inc., Hayward, CA, USA). The microplates were inoculated with a cell suspension at a cell density of 95-96% turbidity and dye IF-A. Further additives were vitamin, micronutrient and sea-salt solutions, which had to be added for dealing with such marine bacteria [24]. The plates were sealed with parafilm to avoid a loss of fluid. The measurement data were exported and further analyzed with the opm package for R [7,25], using its functionality for statistically estimating parameters from the respiration curves such as the maximum height, and automatically translating these values into negative, ambiguous, and positive reactions.

The following substrates were utilized in the Generation-III plates: positive control, pH 6, 1% NaCl, 4% NaCl, D-galactose, D-fucose, L-fucose, L-rhamnose, 1% sodium lactate, D-arabitol, *myo*-inositol, rifamycin SV, L-aspartic acid, L-glutamic acid, L-histidine, L-serine, D-glucuronic acid, quinic acid, L-lactic acid, citric acid, *α*-keto-glutaric acid, D-malic acid, L-malic acid, nalidixic acid and sodium formate.

According to Generation-III plates the strain is negative for dextrin, D-maltose, D-trehalose, D-cellobiose, *β*-gentiobiose, sucrose, D-turanose, stachyose, pH 5, D-raffinose, *α*-D-lactose, D-melibiose, *β*-methyl-D-galactoside, D-salicin, *N*-acetyl-D-glucosamine, *N*-acetyl-*β*-D-mannosamine, *N*-acetyl-D-galactosamine, *N*-acetyl-neuraminic acid, 8% NaCl, D-glucose, D-mannose, D-fructose, 3-O-methyl-D-glucose, inosine, fusidic acid, D-serine, D-sorbitol, D-mannitol, glycerol, D-glucose-6-phosphate, D-fructose-6-phosphate, D-aspartic acid, D-serine, troleandomycin, minocycline, gelatin, glycyl-L-proline, L-alanine, L-arginine, L-pyroglutamic acid, lincomycin, guanidine hydrochloride, niaproof, pectin, D-galacturonic acid, L-galactonic acid-gamma-lactone, D-gluconic acid, glucuronamide, mucic acid, D-saccharic acid, vancomycin, tetrazolium violet, tetrazolium blue, *p*-hydroxy-phenylacetic acid, methyl pyruvate, D-lactic acid methyl ester, bromo-succinic acid, lithium chloride, potassium tellurite, tween 40, *γ*-amino-n-butyric acid, *α*-hydroxy-butyric acid, *β*-hydroxy-butyric acid, *α*-keto-butyric acid, acetoacetic acid, propionic acid, acetic acid, aztreonam, butyric acid and sodium bromate and the negative control.

In a previous study by Suzuki *et al.* [1], bacterial growth on nine substrates was tested for *R. elongatum* OCh 323^T^. According to [1], none of the carbon sources were utilized. In contrast, the OmniLog assay resulted in more than fifteen positive reactions, including sugars, carboxylic and amino acids. This observation can be explained by a higher sensitivity of respiration measurements compared to growth measurements [26]. For instance, the positive reactions detected only in the OmniLog instrument but not by Suzuki *et al.* [1] might be caused by substrates that were only partially metabolized.

### Chemotaxonomy

The principal cellular fatty acids of strain OCh 323^T^ are C_18:1_ (68%), C_16:0_ (12%), C_18:0_ (8%), C_19:0 cyclo_ (4%), C_16:0 2-OH_ (2%), C_14:0 3-OH_ (2%), C_15:0_ (1%), C_17:0_ (1%), and C_16:1_ (1%), whereas C_14:0_ and C_18:2_ are only found in traces (all data from [1]).

## Genome sequencing and annotation

### Genome project history

The genome of strain DSM 19469^T^ was sequenced within the DFG funded project “Ecology, Physiology and Molecular Biology of the *Roseobacter* group: Towards a Systems Biology Understanding of a Globally Important Clade of Marine Bacteria”. The strain was chosen for genome sequencing according the *Genomic Encyclopedia of Bacteria and Archaea* (GEBA) criteria [27,28].

Project information can found in the Genomes OnLine Database [29]. The Whole Genome Shotgun (WGS) sequence is deposited in GenBank and the Integrated Microbial Genomes database (IMG) [30]. A summary of the project information is shown in Table 2.

**Table 2:** Genome sequencing project information

MIGS ID	Property	Term
MIGS-31	Finishing quality	finished
MIGS-28	Libraries used	Two genomic libraries: one Illumina PE library (441 bp insert size), one 454 PE library (3 kb insert size)
MIGS-29	Sequencing platforms	Illumina GA IIx, Illumina MiSeq
MIGS-31.2	Sequencing coverage	93 ×
MIGS-30	Assemblers	Velvet version 1.1.36, Newbler version 2.3, Consed 20.0
MIGS-32	Gene calling method	Prodigal 1.4
	INSDC ID	CP004372
	GenBank Date of Release	*pending publication*
	GOLD ID	Gi21384
	NCBI project ID	189501
	Database: IMG	2522572126
MIGS-13	Source material identifier	DSM 19309^T^
	Project relevance	Tree of Life, biodiversity

### Growth conditions and DNA isolation

A culture of strain DSM 19469^T^ was grown aerobically in DSMZ medium 514 [31] at 28°C. Genomic DNA was isolated using Jetflex Genomic DNA Purification Kit (GENOMED 600100) following the standard protocol provided by the manufacturer but modified by an incubation time of 60 min, incubation on ice over night on a shaker, the use of additional 50 μl proteinase K, and the addition of 100 μl protein precipitation buffer. DNA is available from the DSMZ through the DNA Network [32].

### Genome sequencing and assembly

The genome was sequenced using a combination of two libraries (Table 2). Illumina sequencing was performed on a GA IIx platform with 150 cycles. The paired-end library contained inserts of an average of 441 bp in length. The first run delivered 2.7 million reads. To increase the sequencing depth, a second Illumina run was performed, providing another 1.2 million reads. After error correction and clipping by fastq-mcf [33] and quake [34], the data was assembled using Velvet [35]. The first draft assembly from 1,753,098 filtered reads with an average read length of 89 bp resulted in 97 contigs.

To gain information on the contig arrangement an additional 454 run was performed. The paired-end jumping library of 3kb insert size was sequenced on a 1/8 lane. Pyrosequencing resulted in 174,493 reads, with an average read length of 360 bp, assembled with Newbler (Roche Diagnostics). The resulting draft assembly consisted of 22 scaffolds. Both draft assemblies (Illumina and 454 sequences) were fractionated into artificial Sanger reads 1,000 bp in length plus 75 bp overlap on each site. These artificial reads served as an input for the phred/phrap/consed package [36]. In combination the assembly resulted in 39 contigs organized in four scaffolds. Subsequently, small unlocalized contigs were mapped to the scaffolds using both minimus2 [37] and NUCmer [38]. By manual editing, the number of contigs could be reduced to 21, organized in one chromosomal scaffold. The remaining ordered gaps were closed by bridging PCR fragments and primer walking. A total of 50 reactions were required to conclude the assembly process. The genome was sequenced with a 93× coverage.

### Genome annotation

Genes were identified using Prodigal [39] as part of the JGI genome annotation pipeline. The predicted CDSs were translated and used to search the National Center for Biotechnology Information (NCBI) nonredundant database, UniProt, TIGR-Fam, Pfam, PRIAM, KEGG, COG, and InterPro databases. Identification of RNA genes was carried out by using HMMER 3.0rc1 [40] (rRNAs) and tRNAscan-SE 1.23 [41] (tRNAs). Other non-coding genes were predicted using INFERNAL 1.0.2 [42] Additional gene prediction analysis and functional annotation was performed within the Integrated Microbial Genomes - Expert Review (IMG-ER) platform [43] CRISPR elements were detected using CRT [44] and PILERCR [45].

## Genome properties

**Figure 3:**
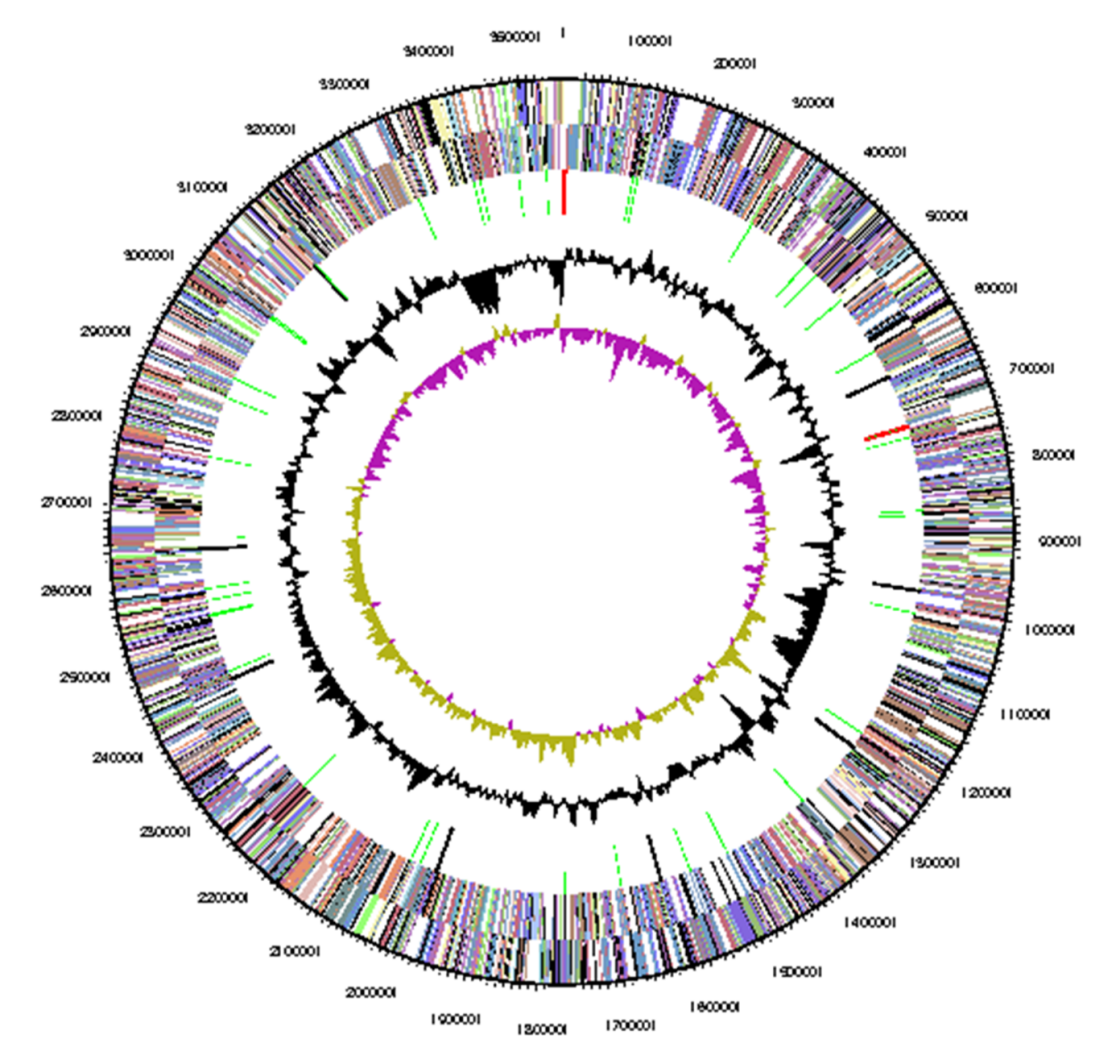
Graphical map of the chromosome. From outside to center: Genes on forward strand (colored by COG categories), Genes on reverse strand (colored by COG categories), RNA genes (tRNAs green, rRNAs red, other RNAs black), GC content (black), GC skew (purple/olive).

The genome statistics are provided in Table 3 and Figure 3. The genome has a total length of 3,555,109 bp and a G+C content of 65.7%. Of the 3,599 genes predicted, 3,540 were identified as protein-coding, and 59 as RNAs. The majority of the protein-coding genes were assigned a putative function (79.6%) while the remaining ones were annotated as hypothetical proteins. The distribution of genes into COG functional categories is presented in Table 4.

**Table 3:** Genome Statistics

**Attribute**	Value	% of Total
Genome size (bp)	3,555,109	100.00
DNA coding region (bp)	3,196,272	89.91
DNA G+C content (bp)	2,337,214	65.74
Number of scaffolds MIGS-9	1	
Extrachromosomal elements MIGS-10	0	
Total genes	3,599	100.00
RNA genes	59	1.64
rRNA operons	2	
tRNA genes	45	1.25
Protein-coding genes	3,540	98.36
Genes with function prediction (proteins)	2,864	79.58
Genes in paralog clusters	2,870	79.74
Genes assigned to COGs	2,750	76.41
Genes assigned Pfam domains	2,942	81.74
Genes with signal peptides	291	8.09
Genes with transmembrane helices	827	22.98
CRISPR repeats	0	

**Table 4:** Number of genes associated with the general COG functional categories

**Code**	**Value**	**%age**	**Description**
J	164	5.5	Translation, ribosomal structure and biogenesis
A	0	0.0	RNA processing and modification
K	149	5.0	Transcription
L	133	4.4	Replication, recombination and repair
B	4	0.1	Chromatin structure and dynamics
D	25	0.8	Cell cycle control, cell division, chromosome partitioning
Y	0	0.0	Nuclear structure
V	32	1.1	Defense mechanisms
T	88	2.9	Signal transduction mechanisms
M	191	6.4	Cell wall/membrane/envelope biogenesis
N	35	1.2	Cell motility
Z	1	0.0	Cytoskeleton
W	0	0.0	Extracellular structures
U	52	1.7	Intracellular trafficking and secretion, and vesicular transport
O	124	4.1	Posttranslational modification, protein turnover, chaperones
C	217	7.3	Energy production and conversion
G	174	5.8	Carbohydrate transport and metabolism
E	338	11.3	Amino acid transport and metabolism
F	83	2.8	Nucleotide transport and metabolism
H	143	4.8	Coenzyme transport and metabolism
I	134	3.5	Lipid transport and metabolism
P	167	5.6	Inorganic ion transport and metabolism
Q	90	3.0	Secondary metabolites biosynthesis, transport and catabolism
R	379	12.7	General function prediction only
S	272	9.1	Function unknown
-	849	23.6	Not in COGs

## Genomic insights

### Genome size, genome comparisons and extrachromosomal elements

Whole genome sequencing of strain *R. elongatum* DSM 19469^T^ revealed a complete and finished genome size of 3,555,109 bp, which seems to be the smallest completed genome of representatives of the *Roseobacter* group up to date [46]. The two other isolates *Loktanella vestfoldensis* SKA53 and *Sulfitobacter* sp. EE-36 both reveal a genome length shorter than that of strain DSM 19469^T^, but remain still in draft state. Whereas many members of the *Roseobacter* group contain plasmids [47], no extrachromosomal elements could be detected in strain DSM 19469^T^.

**Figure 4:**
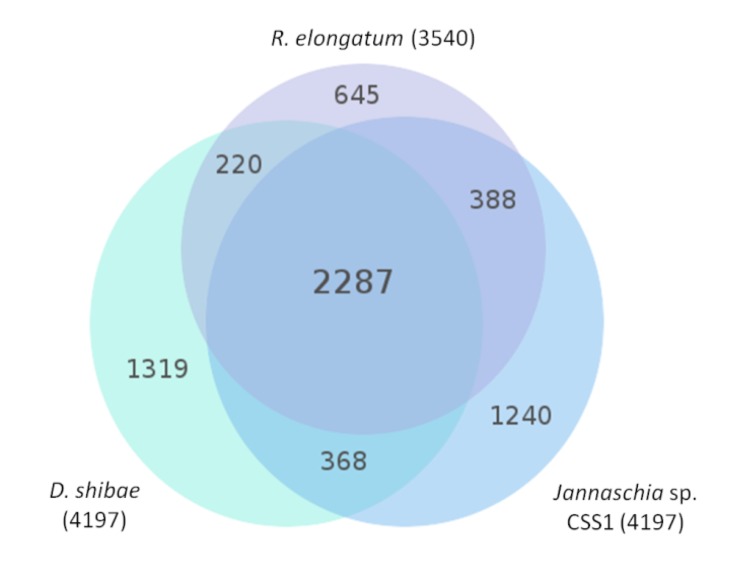
Venn diagram (total numbers in parentheses) of *R. elongatum* DSM 19469^T^, *D. shibae* DSM 16493^T^ and *Jannaschia* sp. CSS1.

The fraction of shared genes between strain *R. elongatum* DSM 19469^T^ and the neighboring strains *D. shibae* DFL-12 (DSM 16493^T^) [11,48] (Figure 1) and *Jannaschia* sp. CSS1 (which turned out to have similar genomic characteristics in the course of this study, too), both members of the *Roseobacter* group are shown in a Venn diagram (Figure 4). The number of pairwise genes was inferred from the phylogenetic profiler of the IMG-ER platform [43]. Homologous genes were detected with an E-value cutoff of 10^-5^ and a minimum identity of 30%.

A total of 2,287 genes are shared by all three genomes, corresponding to 54.3% and 53.4% of the gene count in *D. shibae* DSM 16493^T^ [11,48] and *Jannaschia sp.* CCS1, respectively. With only 3.5 Mbp in length, the genome of *R. elongatum* DSM 19469^T^ shares more than 64.6% of genes with the other two genomes. A number of 645 genes that have no homologs in the other genomes were detected, including a sensor protein of blue-light using FAD (BLUF, roselon_02123) and the Phn gene cluster (roselon_02168-79) involved in the uptake and degradation of phosphonates.

### Phages

Phages are widely distributed and common in marine environments [49-51]. Horizontal gene transfer of the phage genome and its integration in the host genome are known to drive the bacterial diversity [51,52]. In the genome sequence of *R. elongatum* DSM 19469^T^ several putative phage-associated gene sequences were detected, particularly organized in gene clusters (e.g., roselon_02355 - 02370).

### Quorum sensing

Quorum sensing (QS) is a cell-to-cell communication system, where bacteria interact with each other in dependence of their population density. Gram-negative bacteria use small signal molecules called autoinducers, which are produced, excreted through the bacterial membrane and detected by conspecific bacteria. Consequently, when the concentration of those membrane-diffusible autoinducers reaches a specific threshold value, the population responds with an activation of gene expression to coordinate a population-wide behavior [53-58]. QS was first detected in the marine gammaproteobacterium *Vibrio fischerii*, a species often found to live in symbiosis with squids or fishes. Here, the autoinducer accumulation and the activation of certain genes result in biolumescence [59,60]. Other examples for QS-induced bacterial physiological aspects are biofilm formation, exopolysaccharide production and virulence [53,61]. Interestingly, many representatives of the *Roseobacter* group were shown to encode and/or express gene sequences associated with QS [e.g., 62-65].

Genome analysis of strain *R. elongatum* DSM 19469^T^ revealed the presence of genes putatively associated with QS like a N-acyl-L-homoserine lactone synthetase (*LuxI* homolog; roselon_01555) and a regulator of the *LuxR* family (roselon_3097).

### Photosynthetic gene cluster

Light is used as energy source by many bacteria in the ocean. An increasing number of representatives belonging to the *Roseobacter* group have been found to be aerobic anoxygenic photoheterotrophs, containing bacteriochlorophyll *a* (Bchl *a*) [3,4,66-69]. They transform light energy into a proton motive force (*pmf*) across the membrane that is used for the generation of ATP, which could have an importance for marine environments and global cycles [66-68]. Aerobic anoxygenic photoheterotrophs represent a significant fraction of the microbial population depending on the location [69-73]. It was further shown that aerobic anoxygenic photoheterotrophs synthesize Bchl *a* only in the presence of oxygen [66,74] and that the photosynthetic pigments of aerobic alphaproteobacteria are synthesized under dark conditions [75-77], whereas some members of the gammaproteobacterial OM60/NOR5 clade also synthesize pigments in the light [78]. Furthermore, Elsen and colleagues reported that genes encoding the photosynthetic apparatus and related genes are mainly organized in a large gene cluster [79].

In the description of strain OCh 323^T^, the authors showed that the absorption spectrum of the membranes of ultrasonically disrupted cells exhibit a significant photosynthetic reaction center absorption peak (at 800 nm) and a light-harvesting complex I absorption peak (at 879 nm) [1].

**Figure 5:**
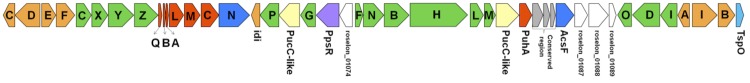
Arrangement of the photosynthetic gene cluster. Green, *bch* genes; red, *puf* genes; orange, *crt* genes; blue, *hem* genes; purple, genes for sensor proteins, white, other genes (adapted after [77,80]).

The genome sequence of strain *R. elongatum* DSM 19469^T^ encodes a functional photosynthetic gene cluster (roselon_01064 - 01096) containing a set of *bch* genes, *puf* genes, *crt* genes, *hem* genes and genes for proteins with sensory activity (Figure 5).

### Motility and flagellar genes

Strain *R. elongatum* DSM 19469^T^ was originally described as non-flagellated [1]. In the genome a flagella gene cluster was found flanking the chromosome-partitioning gene *dna*A (roselon_1273). Flagella formation depends on external stimuli such as incubation temperature or composition of the media [81]. Thus, strain DSM 19469^T^ might exhibit a motile phenotype under certain, as yet unknown, conditions. Flagellar genes of strain DSM 19469^T^ involved in flagellar assembly and function were analyzed to assess potential motility behavior. The cluster consists of 28 genes (roselon_01279 - 01316). Three further motor switch proteins, including *fli*G were detected upstream of roselon_03222. Together with *fli*M (roselon_03295) and *fli*N (roselon_01309) *fli*G forms a protein that controls rotation behavior of flagella. This dissociation of flagellar operons has been seen in two groups of alphaproteobacteria [82]. No master regulator genes operon (*flh*DC) [83] could be detected. Whereas genes controlling the early flagellum assembly were not detected, several proteins necessary for the formation of the basal body were found, including *flg*DEFGHIKL and *fli*F. Genome analysis of strain DSM 19469^T^ revealed further the presence of genes involved in the formation of the export apparatus: the previously mentioned C-ring forming complex *fli*GMN and the protein-encoding sequences *flh*A, *flh*B, *fli*P, *fli*Q and *fli*R, which are involved in pore-forming through the membrane [84]. Whereas two motor protein-encoding gene sequences *mot*AB were found (roselon_01316, roselon_01313), a homolog of the *fli*O gene as part of the channel-forming apparatus was absent. Additionally, the genome of strain *R. elongatum* DSM 19469^T^ revealed the presence of regulatory genes controlling the late phase, such as the hook capping protein (roselon_01279), the flagellar hook-length control protein (roselon_01280) and the flagellin-encoding gene sequence *fli*C (roselon_01284). Methyl-accepting chemotaxis proteins that sense external stimuli, and therefore direct flagella-induced motility of strain DSM 19469^T^, could not be detected.

**Figure 6:**
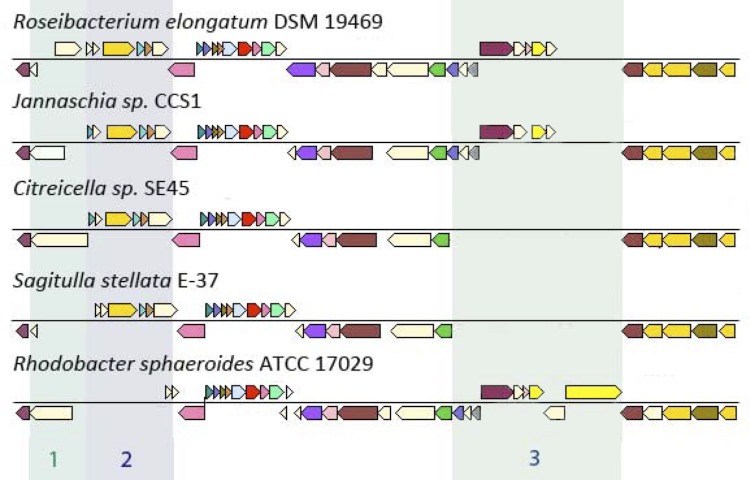
Map of the flagella cluster of *R. elongatum* DSM 19469^T^ (roselon_Rosei_p5_w02) and homologous ORFs in the genomes of the four comparable strains *Jannaschia* sp. CCS1 (NC_007802), *S. stellata* E-37 (NZ_AAYA01000005), *R. sphaeroides* ATCC 17029 (NC_009049) and *Citreicella* sp. E45 (NZ_GG704601). Prediction of homologs was conducted using the conserved-neighborhood tool of the IMG-ER platform [43]. The colored areas represent differences in the genomic structure within the flagella cluster.

To compare the flagellar gene clusters of neighboring species (Figure 6), homologs of *flg*G coding for a protein mainly involved in the formation of the basal body in *R. sphaeroides* ATCC 17029 [85] were identified using the IMG/ER platform [43]. All compared genomes show a similar gene cluster structure, but have variations such as differences in gene length for *fli*K, which controls the completion of previous flagellum-assembly steps. The *fli*K protein in *R. sphaeroides* is 700 amino-acid residues (AA) in length [85]. A genome BLAST search (minimal similarity 30%, maximal e-value 10^-5^) against putative *fli*K proteins revealed that the gene-encoding sequence length of *fli*K varies from 102 AA in *R. sphaeroides* strains WS8N and 2.4.1 to 937 AA in *Citreicella* sp. SE45. The genomes of the three species *Salipiger mucosus*, *Sagittulla stellata* and *Pelagibaca bermudensis* each encode a truncated *fli*K-encoding gene sequence, but those strains do not form flagella [86-88]. These truncations could be the reason for inactive proteins resulting in a non-motile phenotype. In contrast, the genome of *Jannaschia* sp. CCS1 codes for a *fli*K protein of 612 AA (Jann_4206) and, interestingly, this strain was reported to be motile.

The second marked region (Figure 6) is well conserved in the first four genomes, but is missing in strain *R. sphaeroides* ATCC 17029. This cluster consists of the rod-forming gene *flg*J and three proteins involved in the regulation of the flagella assembly. Homologs of the *R. elongatum* DSM 19469^T^ flagellin gene (roselon_01284) are absent in *R. sphaeroides*. Thus, the regulation of the flagella operon might be conducted by other genes: one of the genes coding for the flagellin-forming FliC in *R. sphaeroides* is located on the chromosome within the flagellar cluster. An additional set of three regulation genes is detected on the 120 kb plasmid (NC_009040) of the genome. In area 3 of Figure 6 the genomes of both *S. stellata* and *Citreicella sp.* lack three flagellar genes: *fli*L *and fli*F, which are both involved in the formation of the basal body, and *fli*P (export apparatus). An additional PAS/PAC sensor hybrid histidine kinase (Rsph17029_2967) is found in the *R. sphaeroides* genome.

### Morphological traits

The genome sequence of strain *R. elongatum* DSM 19469^T^ was found to have specific genes associated with the putative biosynthesis and export of exopolysaccharides (roselon_01150, roselon_01343 - 01343) and the putative export of capsule polysaccharides (e.g., roselon_00513, roselon_01783 - 01785).

Additionally, the genome of strain *R. elongatum* DSM 19469^T^ encodes several gene sequences associated with flp-type pili biogenesis and formation (e.g., roselon_01843 - 01852). Hence, the formed pili might play a role in adhesion or switching-type motility on solid surfaces.

Further, strain *R. elongatum* DSM 19469^T^ seems to accumulate polyhydroxyalkanoates as storage compounds (e.g., roselon_00211 - 00214).

### Metabolic plasticity

The genome sequence of strain *R. elongatum* DSM 19469^T^ encodes a gene cluster associated with a Sox multienzyme complex (roselon_02191 - 02202) that could be utilized for the oxidation of thiosulfate to sulfate. Carbon monoxide could be putatively oxidized by aerobic-type carbon monoxide dehydrogenases (roselon_01738, roselon_01976 - 01977, roselon_02472, roselon_02474).

Several genes play a role in the electron transport chain, such as those associated with the NADH dehydrogenase (e.g., roselon_00011 - 00023), succinate dehydrogenase (roselon_01681 - 01684) and cytochrome *bd* ubiquinol oxidase (roselon_00027 - 00028). In addition two different cytochrome *c* oxidases (*caa*_3_-type [e.g. roselon_02733 - 02734] or *cbb*_3_-type [roselon_00626 - 00628]) were detected.
